# Growth, phytohormone and transcriptome responses of *Cunninghamia lanceolata* seedlings to different light qualities

**DOI:** 10.3389/fpls.2026.1765282

**Published:** 2026-04-07

**Authors:** Bo Liu, Zhengning Wang, Yushuang Song, Qingqing Liu, Lixin Wang

**Affiliations:** 1 Shandong Key Laboratory of Wetland Ecology and Biodiversity Conservation in the Lower Yellow River, College of Life Sciences, Qufu Normal University, Qufu, Shandong, China; 2 School of Soil and Water Conservation, Jiangxi University of Water Resources and Electric Power, Nanchang, Jiangxi, China; 3 Department of Earth and Environmental Sciences, Indiana University Indianapolis, Indianapolis, IN, United States

**Keywords:** Chinese fir, conifer seedlings, far-red light, light spectra, shade adaptation

## Abstract

Light is the primary energy source for photosynthesis and an important signal regulating seedling growth, hormonal balance, and metabolic adaptation. In this study, seedlings of *Cunninghamia lanceolata* were grown under six light quality treatments, including white light (WL), blue light (BL), red light (RL), far-red light (FrL), and mixed RL: FrL ratios at 1:1 (1:1L) and 1:2 (1:2L). All treatments were applied at a constant photosynthetic photon flux density of 400 μmol·m^-2^·s^-1^. The results showed that BL, FrL, and 1:2L significantly promoted stem elongation, whereas RL and 1:1L increased root collar diameter. Hormone assays revealed a clear antagonistic pattern between gibberellic acid (GA_3_) and abscisic acid (ABA), with GA_3_ contents elevated under BL and FrL but reduced under RL, while ABA showed the opposite trend. Transcriptomic analysis showed that different light spectra regulated the expression of photoreceptors (PHYB, CRY1 and CRY2) and downstream factors (COP1, PIF3, HY5). BL stimulated flavonoid biosynthesis, RL enhanced phenylpropanoid and cell wall related pathways, and FrL induced carotenoid and stilbenoid metabolism. There results indicated that light spectra regulate seedling growth by integrating photoreceptor signaling, hormone dynamics, and secondary metabolism. The study improves our understanding of light responses in gymnosperms and provides a physiological and molecular basis for optimizing nursery light management and afforestation practices.

## 1 Introduction

Light not only provides the energy for photosynthesis but also acts as a crucial environmental signal regulating plant growth, development, and stress adaptation (Doneva et al., 2024; Wang et al., 2024). Plants perceive light mainly through photoreceptors such as phytochromes and cryptochromes, which in turn regulate downstream signaling factors including COP1, PIFs, and HY5. Through these regulatory cascades, light perception is integrated with transcriptional programs to coordinate photomorphogenesis (Toman et al., 2024). For example, red light activates phyB, leading to PIF3 degradation and COP1 inhibition, which enhances HY5-dependent photomorphogenesis and suppresses excessive elongation (Toman et al., 2024). Conversely, far-red light mainly activates phyA, initiating shade avoidance responses such as stem elongation and altered biomass allocation (Sellaro et al., 2025).

Beyond photoreceptor signaling, light strongly interacts with phytohormones and metabolic pathways to shape growth responses (Lauria et al., 2024; Liu et al., 2024). Transcription factors such as PIFs and HY5 function as key nodes linking light with hormone pathways (Matsuo et al., 2019). The balance between growth promoting hormones such as gibberellins (GAs) and auxin, and growth-inhibiting hormones such as abscisic acid (ABA), is particularly important for adaptive morphogenesis under fluctuating light conditions (García-Martinez and Gil, 2001; Weyers and Paterson, 2001). Light quality also modulates secondary metabolism; for instance, blue light enhances flavonoid biosynthesis, which regulates auxin transport and contributes to stress tolerance (Lauria et al., 2024; Van Brenk et al., 2025). Thus, these studies emphasize the interplay of light, hormones, and metabolism in determining plant developmental strategies.

Although substantial progress has been made in model species and horticultural crops (Boros et al., 2023), studies on light quality effects in woody plants, particularly gymnosperms, remain limited (OuYang et al., 2015). As typical gymnosperms, conifers possess large and relatively conserved genomes, which may underlie unique light response mechanisms compared with angiosperms (Wan et al., 2022). Conifers often accumulate flavonoids, terpenes, and other secondary metabolites to enhance stress tolerance under shaded environments (OuYang et al., 2015). However, direct molecular evidence for light quality dependent regulation of these strategies in gymnosperms is lacking.

The light environment in the understory is highly heterogeneous and dynamic due to reflection, absorption, and filtering of solar radiation by the canopy (Wirth et al., 2001; Montgomery and Chazdon, 2002). A typical feature is reduced RL, caused by chlorophyll absorption, and enrichment of FrL, leading to a low red:far-red (R:Fr) ratio (Huber et al., 2021). Typically, the R:Fr ratio is about 1.2 in open sunlight but can decline to 0.2 beneath dense canopies (Ruberti et al., 2012). This spectrum is a key driver of shade responses, regulating seedling elongation growth, biomass allocation, and survival strategies (Gómez-Ocampo et al., 2023; Kang et al., 2024; Li et al., 2025). While most studies have focused on white, blue, or red light, the ecological roles of far red and variable red-to-far red ratios have received far less attention, despite their particular relevance under natural canopy conditions (Wei et al., 2019, 2023; Navidad et al., 2020).

*Cunninghamia lanceolata* (Chinese fir) is an evergreen conifer widely distributed in subtropical regions of southern China. It is one of the most important plantation tree species for timber production and ecological restoration (Liu et al., 2018). Chinese fir plantations cover more than 17 million hectares in China and account for a substantial proportion of global planted forests, highlighting their major economic and ecological importance (Liu et al., 2018; Yang et al., 2018). The species is generally considered light-demanding during early developmental stages, and its regeneration and seedling growth are strongly influenced by light environment and canopy conditions (Liu et al., 2018). As a representative gymnosperm, its responses to spectral composition may differ from those of angiosperms. Previous studies have examined physiological responses of *C. lanceolata* seedlings to light quality (Wang et al., 2022), but transcriptional mechanisms remain poorly understood (Chen et al., 2025). Existing reports are limited to RL and BL effects on photosynthetic genes (Liu et al., 2022), leaving the molecular interplay between photoreceptor signaling, hormone regulation, and seedling morphogenesis largely unexplored.

In this study, we conducted a controlled experiment with *C. lanceolata* seedlings under six light quality treatments. By integrating RNA sequencing, hormone profiling, and morphological assessments, we aimed to elucidate the regulatory mechanisms underlying light perception, hormonal crosstalk, and metabolic adjustment in this ecologically and economically important conifer. Specifically, our objectives were (1) to assess the effects of light quality on seedling growth and morphological traits; (2) to identify and characterize differentially expressed genes with an emphasis on the transcriptional responses of photoreceptors and their downstream regulators; and (3) to evaluate the antagonistic interaction between gibberellins and abscisic acid under different light conditions.

## 2 Materials and methods

### 2.1 Light experiment design and plant materials

The experiment included six light quality treatments: white light (WL), blue light (BL), red light (RL), far red light (FrL), mixed red and far-red light at a 1:1 ratio (RFr1:1L, hereafter referred to as 1:1L), and mixed red and far-red light at a 1:2 ratio (1:2L). These light-quality treatments were selected to represent ecologically relevant spectral environments and to isolate the roles of specific wavelength regions in regulating seedling growth. In forest understories, canopy filtering reduces red light and enriches far-red radiation, resulting in a decreased red:far-red (R:FR) ratio that strongly influences seedling morphology and development. Therefore, red light, far-red light, and mixed R:FR ratios were included to simulate canopy-filtered light conditions, while blue and red light treatments were used to examine wavelength-specific photoreceptor signaling responses. Similar spectral combinations have been widely applied in previous studies on forest tree seedlings under controlled environments, including *Cunninghamia lanceolata* and other woody species (Liu et al., 2022; Wang et al., 2024). Mixed spectra were generated by combining red and far red light emitting diode LED beads in the designated ratios. For all treatments, the photosynthetic photon flux density (PPFD) was maintained at 400 μmol·m^-2^·s^-1^ and was monitored using an HP350 light meter (HiPoint Inc., Taichung City, Taiwan, China).

The experiment was conducted in independent growth chambers. Each chamber (90 × 100 × 100 cm) was built with a steel frame and covered with light blocking black film to eliminate external light interference. An airflow circulation system was installed at the top of each chamber to stabilize temperature and ensure proper air circulation. Two LED panels (40 × 80 cm) were mounted horizontally at the top of each chamber. During the experiment, as the seedlings grew, the vertical distance between the top of the seedling canopy and LED panels was kept approximately 20 cm in order to ensure uniform lighting conditions. The photoperiod was set to 12 h per day (from 06:00 to18:00) controlled by automatic timer.

One year old clonal seedlings of *C. lanceolata* with uniform and healthy growth were selected. In each chamber six potted seedlings were grown under the assigned light treatment. Seedling pots were treated as experimental replicates. Pots were spaced to avoid mutual shading (approximately 30 cm between pots), randomly positioned within each chamber, and repositioned weekly to minimize positional effects and to ensure that all seedlings experienced similar irradiance and microclimatic conditions. The experiment lasted for three months. No fertilizer was applied during the experimental period. Weeds were removed regularly and seedlings were watered as needed to maintain adequate soil moisture.

### 2.2 Determination of growth traits and phytohormones

Seedling growth traits were measured after three months of light exposure. Stem height was defined as the vertical distance from the soil surface to the apical meristem (precision 0.1 cm). Root-collar diameter (RCD) at the stem base was measured using a digital Vernier caliper (precision 0.01 mm). At harvest, seedlings were separated into roots, stems, and leaves, oven heated at 105 °C for 30 min, and subsequently dried at 80 °C to constant mass. Dry mass of roots, stems, and leaves were recorded, and total biomass was calculated.

Two seedling quality indices were calculated from the morphological measurements. The seedling sturdiness index (SI) was defined as the ratio of height to root collar diameter (RCD) (SI=Height/RCD). The Dickson quality index (DQI), which integrates multiple growth traits, was computed as follows (Dickson et al., 1960):


$$\text{DQI}=\frac{{\text{Seedling}}_{\text{Bio}}}{\frac{\text{Height}}{\text{RCD}}+\frac{{\text{Shoot}}_{\text{Bio}}}{{\text{Root }}_{\text{Bio}}}}$$

where RCD is root collar diameter, Plant_Bio_, Shoot_Bio_, Root_Bio_ are the total biomass in the whole seedling, shoot, and root, respectively.

For hormone assays, freshly collected leaves were flash-frozen in liquid nitrogen and stored at –80 °C. For each light treatment, leaves were sampled from three randomly selected seedlings and each seedling was regarded as one independent biological replicate. Abscisic acid (ABA) and gibberellic acid (GA_3_) were extracted by liquid nitrogen grinding, purified with C18 solid-phase extraction cartridges, and quantified using a RIGOL L3000 high-performance liquid chromatography (HPLC) system. Hormone analyses were performed by Suzhou Comin Biotechnology Co., Ltd. (China).

### 2.3 RNA-seq analysis

At the end of the experiment, current year leaves were sampled from seedlings under each light treatment. For each treatment, three biological replicates were collected, giving a total of eighteen samples. Fresh leaf samples were immediately frozen in liquid nitrogen, wrapped in aluminum foil and stored at - 80 °C until RNA extraction.

Total RNA was extracted using the TRIzol reagent (Tiangen Biotech, Beijing, China). RNA concentration and purity were measured with a NanoDrop spectrophotometer, and RNA integrity was assessed using an Agilent 2100 Bioanalyzer. High quality RNA was used for mRNA enrichment with Oligo dT attached magnetic beads. The enriched mRNA was fragmented and reverse transcribed to first stranded cDNA, followed by second strand synthesis with DNA polymerase I. Double stranded complementary DNA was purified with AMPure XP beads, end repaired, adenylated, ligated to sequencing adapters, size selected and amplified by polymerase chain reaction to construct complementary DNA libraries. Library quality and insert size were checked with an Agilent 2100 Bioanalyzer and quantitative polymerase chain reaction. Sequencing was carried out on an Illumina NovaSeq 6000 platform using paired end reads of one hundred and fifty base pairs.

Raw reads were filtered to remove adapters sequences, reads containing unknown bases and low quality reads. Clean reads from all samples were assembled *de novo* using Trinity. Redundant transcripts were clustered with CD HIT and assembly completeness was evaluated with BUSCO and TransRate. The longest open reading frame in each transcript was predicted with TransDecoder. Protein coding sequences were annotated against public databases including NR, NT, KO, Swiss Prot, Pfam, GO and KOG using BLASTp. For each gene, the longest transcript was taken as the unigene reference. Gene level raw read counts estimated by RSEM were used for differential expression analysis and fragments per kilobase of transcript per million mapped reads FPKM were calculated for visualization of expression patterns of selected genes.

### 2.4 Statistical analysis and functional enrichment

Growth traits and phytohormone data were analyzed using one-way analysis of variance (ANOVA), followed by the Least Significant Difference (LSD) test at a significance level of α = 0.05. All statistical analyses were conducted with SPSS v20.0 (IBM, USA), and figures were generated using Origin v 9.1 (OriginLab, USA).

Differentially expressed genes (DEGs) were identified using DESeq2 (Love et al., 2014) based on normalized read counts. Genes with an adjusted p-value (padj) < 0.05 and an absolute log_2_ fold change ≥ 1 were considered significantly differentially expressed.

Functional annotation of the assembled transcripts was performed by BLAST searches against public databases (NR, Swiss-Prot, GO, and KEGG) using an e-value threshold of 1e−5. Functional enrichment analysis of DEGs was conducted at both the Gene Ontology (GO) and Kyoto Encyclopedia of Genes and Genomes (KEGG) levels. GO enrichment analysis was performed using GOseq, which corrects for gene length bias, and KEGG pathway enrichment analysis was conducted using KOBAS 3.0. Enriched terms with an adjusted p-value < 0.05 were considered statistically significant.

## 3 Results

### 3.1 Effects on seedling traits

**Figure 1:**
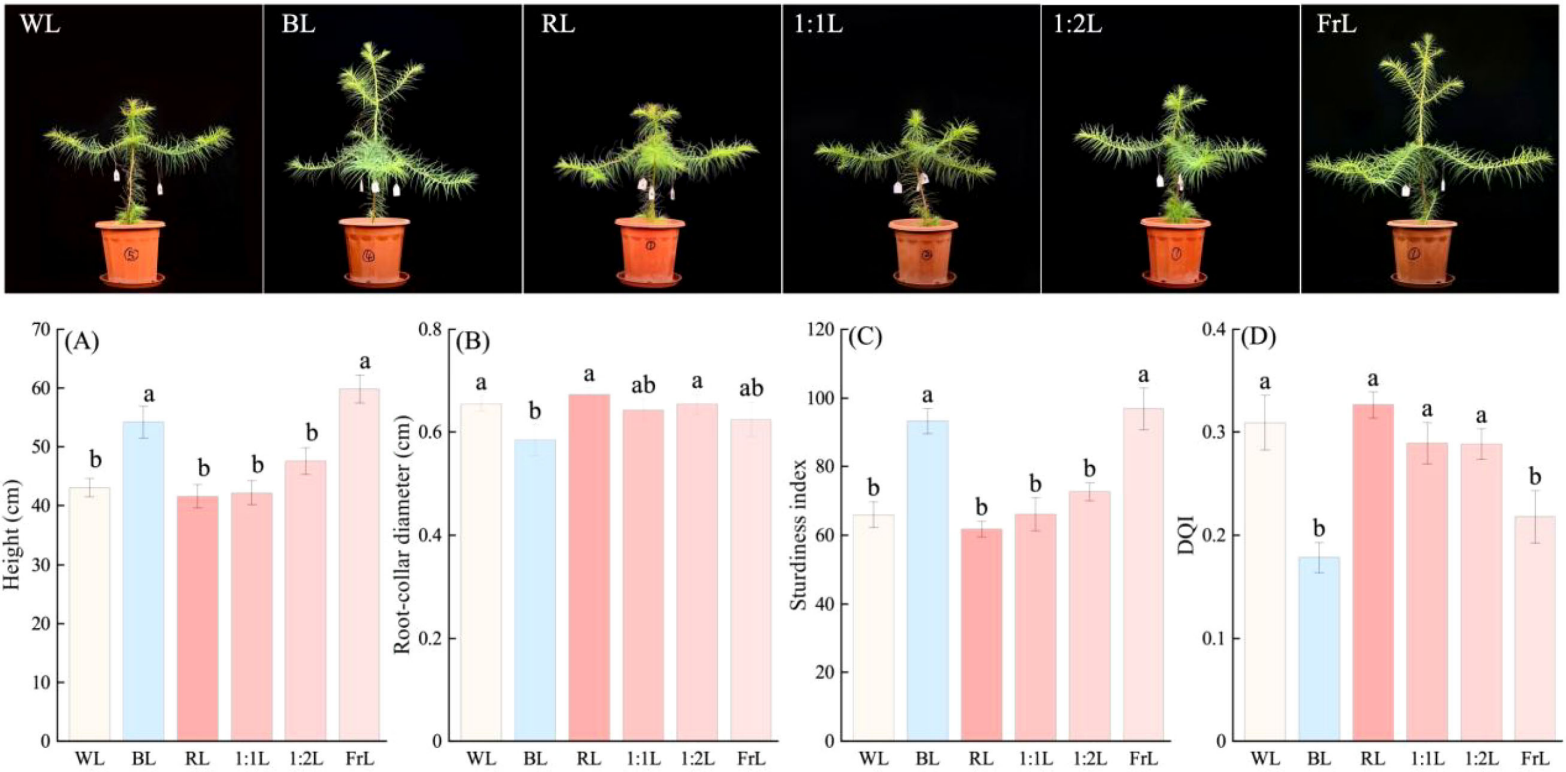
Effects of different light quality treatments on the growth and quality of *C. lanceolata* seedlings. **(A)** Height, **(B)** Root-collar diameter, **(C)** Sturdiness index, and **(D)** Dickson quality index (DQI). Values are means ± standard error (n = 6). Different lowercase letters indicate significant differences among treatments at p < 0.05. Light treatments: WL, white light; BL, blue light; RL, red light; 1:1L, red to far-red ratio 1:1; 1:2L, red to far-red ratio 1:2; FrL, far-red light.

Light quality significantly influenced the morphological development of seedlings (**Figure 1**). Compared to the WL, seedling height was significantly promoted under BL and 1:2L treatments (*p* < 0.05), whereas RL, FrL and 1:1L showed no significant effects (**Figure 1A**). Seedlings exposed to RL resulted in the greatest root-collar diameter, while those under BL exhibited the smallest values (**Figure 1B**). The sturdiness index SI was largest under BL and FrL, indicating that BL and FrL tended to produce more slender seedlings (**Figure 1C**). The Dickson quality index (DQI) of seedlings under RL, 1:1L, and 1:2L treatments similar to white light, and significantly higher than under BL and FrL, with BL and FrL resulting in the lowest DQI values (**Figure 1D**).

### 3.2 Transcriptome sequencing and sequence assembly

Transcriptome sequencing of *C. lanceolata* seedlings under six light quality treatments produced a total of 170.06 Gb of clean data. The data volume per sample ranged from 8.30 Gb (BL) to 11.50 Gb (FrL), corresponding to 55.00 million to 76.26 million clean reads, respectively (**Supplementary Table 1**).

Sequencing quality was consistently high with an average error rate of 0.02%. The mean Q20 and Q30 scores were 98.73% and 96.08%, respectively, suggesting excellent base-calling accuracy. GC content was stable, ranging between 44.27% (RL) and 44.57% (1:2L), with an average of 44.42% (**Supplementary Table 1**).

### 3.3 Differential gene expression analysis

**Figure 2:**
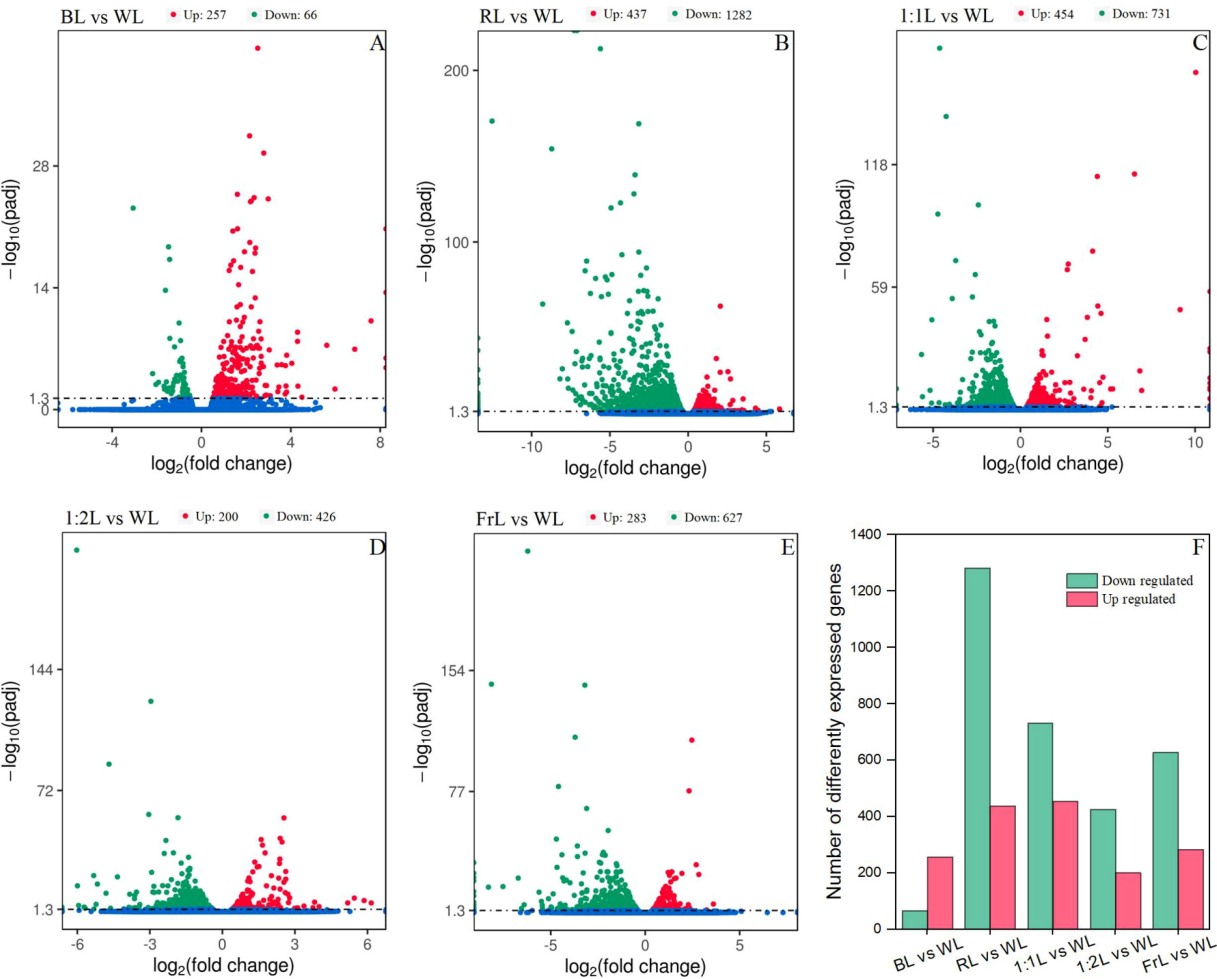
Differentially expressed genes (DEGs) in *C. lanceolata* seedlings under different light quality treatments. **(A–E)** Volcano plots of DEGs between each light treatment (BL, RL, 1:1L, 1:2L, FrL) and WL. The x-axis represents the log2 fold change in gene expression, and the y-axis represents the statistical significance (-log10 adjusted p-value). Red and green dots indicate significantly upregulated and downregulated genes, respectively, while blue dots represent non-significant genes. **(F)** The number of upregulated and downregulated genes identified in each treatment compared with WL.

Substantial differences in gene expression profiles were detected among the light quality treatments (**Figure 2**). BL produced the fewest DEGs 323, most of which were upregulated (257) (**Figures 2A, F**). RL produced the largest number of DEGs compared with WL, with 1,719 DEGs in total, including 437 upregulated and 1,282 downregulated genes (**Figures 2B, F**).

Among the mixed R:Fr treatments, 1:1L generated the greatest number of upregulated genes (454), while1:2L produced the fewest (200) (**Figures 2C, D, F**). FrL treatment resulted in an intermediate response, with 910 DEGs (283 upregulated and 627 downregulated) (**Figures 2E, F**).

### 3.4 GO analysis of DEGs

**Figure 3:**
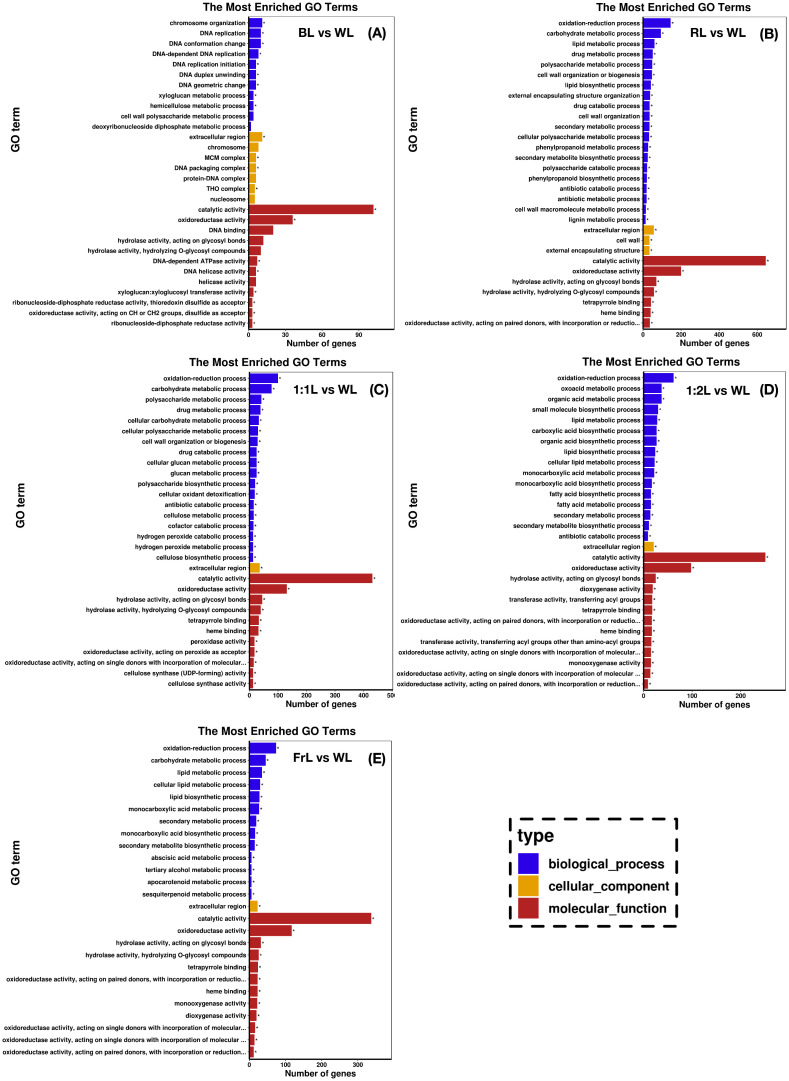
Gene Ontology (GO) enrichment analysis of differentially expressed genes (DEGs) in C. lanceolata seedlings under different light quality treatments: **(A)** BL vs WL, **(B)** RL vs WL, **(C)** 1:1L vs WL, **(D)** 1:2L vs WL, and **(E)** FrL vs WL. Only significantly enriched GO terms are shown. The y-axis represents enriched GO terms, and the x-axis indicates the number of DEGs assigned to each term. Colors distinguish the three GO categories: biological process (blue), cellular component (yellow), and molecular function (red). Asterisks indicate significantly enriched terms (adjusted p < 0.05) based on GOseq analysis.

GO enrichment analysis showed that DEGs were mainly associated with biological processes, particularly “oxidation-reduction process” and “carbohydrate metabolic process” (**Figure 3**). Under BL, 21 GO terms were significantly enriched, with “catalytic activity” and “oxidoreductase activity” representing the most prominent molecular function categories (**Figure 3A**). In addition, processes related to genome stability, such as “chromosome organization” and “DNA replication,” were notably enriched. In RL treatment, enrichment was much broader, with 144 GO terms significantly represented. Key pathways included “carbohydrate metabolic process” and “hydrolase activity, acting on glycosyl bonds,” highlighting the role of RL in modulating carbohydrate decomposition and energy metabolism (**Figure 3B**). For mixed red:far-red treatments, 1:1L showed enrichment in “organic acid metabolic process” and “lipid metabolic process” (**Figure 3C**), while 1:2L further emphasized lipid-related and secondary metabolic processes (**Figure 3D**). Under FrL, DEGs were significantly enriched in hormone-related and pigment-related pathways, particularly “abscisic acid metabolic process” and “carotenoid metabolic process” (**Figure 3E**). These results suggest that light quality shifts differentially regulate primary metabolism, genome maintenance, and secondary metabolite synthesis, thereby shaping light-adaptation strategies in *C. lanceolata* seedlings.

To complement the GO based analysis, KEGG pathway enrichment of the same DEG sets was alsoperformed. DEGs under BL were mainly assigned to flavonoid related and circadian rhythm related pathways, DEGs under RL were concentrated in phenylpropanoid related and cutin, suberin and wax related pathways and DEGs under the mixed red to far red treatments and FrL were enriched in pathways associated with specialized secondary metabolism and protein processing in the endoplasmic reticulum. Detailed KEGG enrichment results, including the full list of significantly enriched pathways for each treatment, are provided in the supplementary material **Supplementary Figure 1**.

### 3.5 Expression of photomorphogenesis-related genes

**Figure 4:**
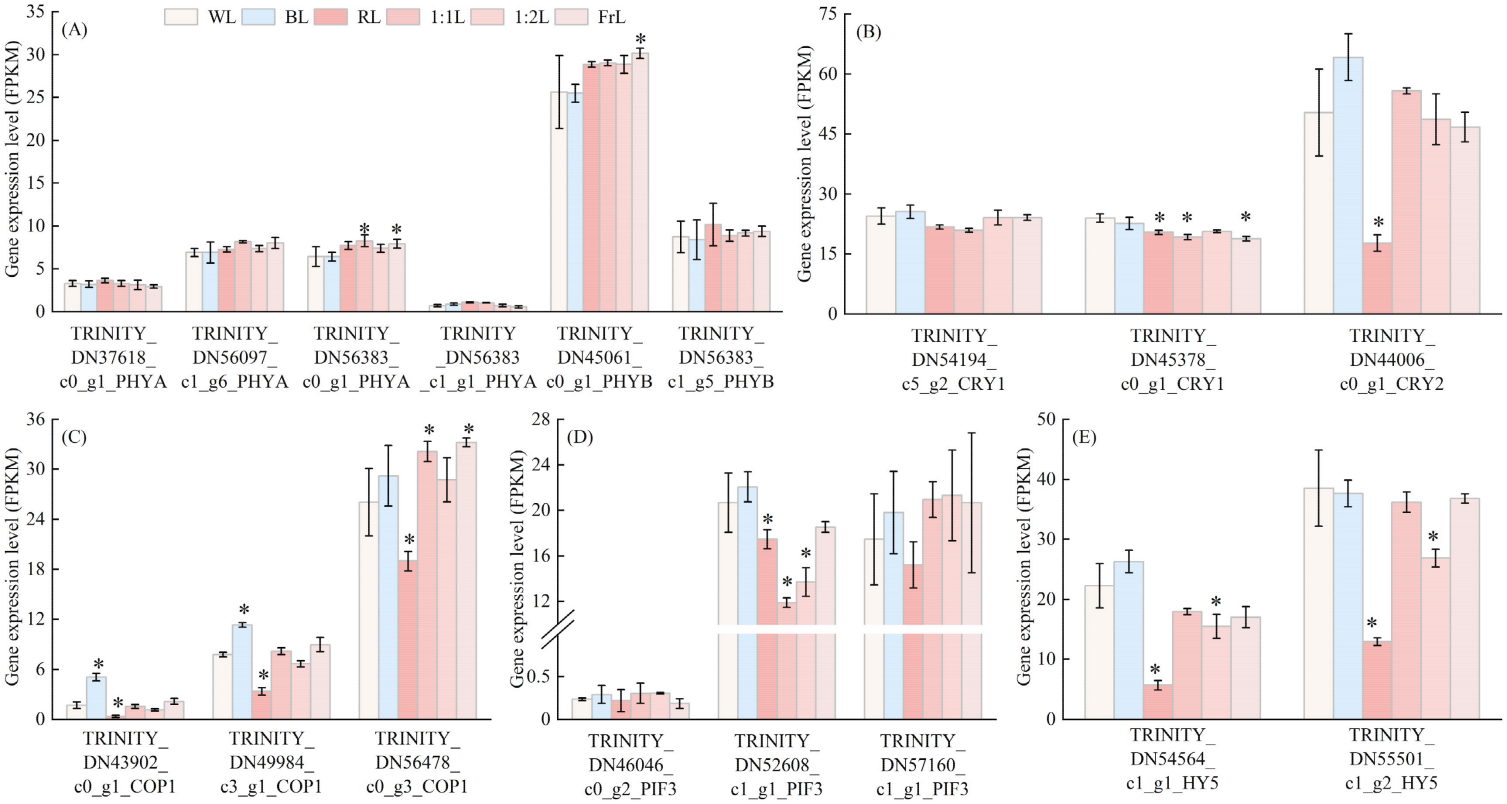
Expression profiles of photomorphogenesis-related genes in *C. lanceolata* seedlings under different light quality treatments. **(A)** PHYA and PHYB, **(B)** CRY1 and CRY2, **(C)** COP1 homologs, **(D)** PIF3 homologs, and **(E)** HY5 homologs. Data represent mean ± standard deviation. * indicates a significant difference compared with WL.

The four PHYA transcripts exhibited consistently low expression levels (FPKM < 10) across all light quality treatments, with no significant differences among treatments (**Figure 4A**). By contrast, the PHYB homolog (TRINITY_DN45061_c0_g1_PHYB) showed strong light quality dependence, reaching its highest expression under RL, which was higher than that under WL and BL. Furthermore, PHYB expression increased progressively with rising FrL ratios (1:1L to FrL) (**Figure 4A**). For the cryptochrome family, two CRY1 homologs (TRINITY_DN54194_c5_g2_CRY1 and TRINITY_DN45378_c0_g1_CRY1) were significantly downregulated under RL and 1:1L treatments. In contrast, CRY2 (TRINITY_DN44006_c0_g1_CRY2) exhibited peak expression under BL but was strongly suppressed under RL (**Figure 4B**). Among ubiquitin ligase genes, three COP1 homologs (TRINITY_DN43902_c0_g1_COP1, TRINITY_DN49984_c3_g1_COP1, and TRINITY_ DN56478_c0 _g3_COP1) were strongly upregulated under BL, but downregulated under RL treatment (**Figure 4C**). For transcription factors, one PIF3 homolog (TRINITY_DN46046_c0_g2_PIF3) showed negligible expression across treatments. In contrast, the other two homologs (TRINITY_DN52608_c1_g1_PIF3 and TRINITY_DN57160_c1_g1_PIF3) exhibited peak expression under BL and FrL, while being significantly repressed under RL (**Figure 4D**). The key positive regulator HY5 (TRINITY_DN54564_c1_g1_HY5 and TRINITY_DN55501_c1_g2_HY5) exhibited the lowest transcript levels under RL treatment, indicating significant suppression by RL (**Figure 4E**).

### 3.6 DEGs in phytohormone signal transduction pathways

**Figure 5:**
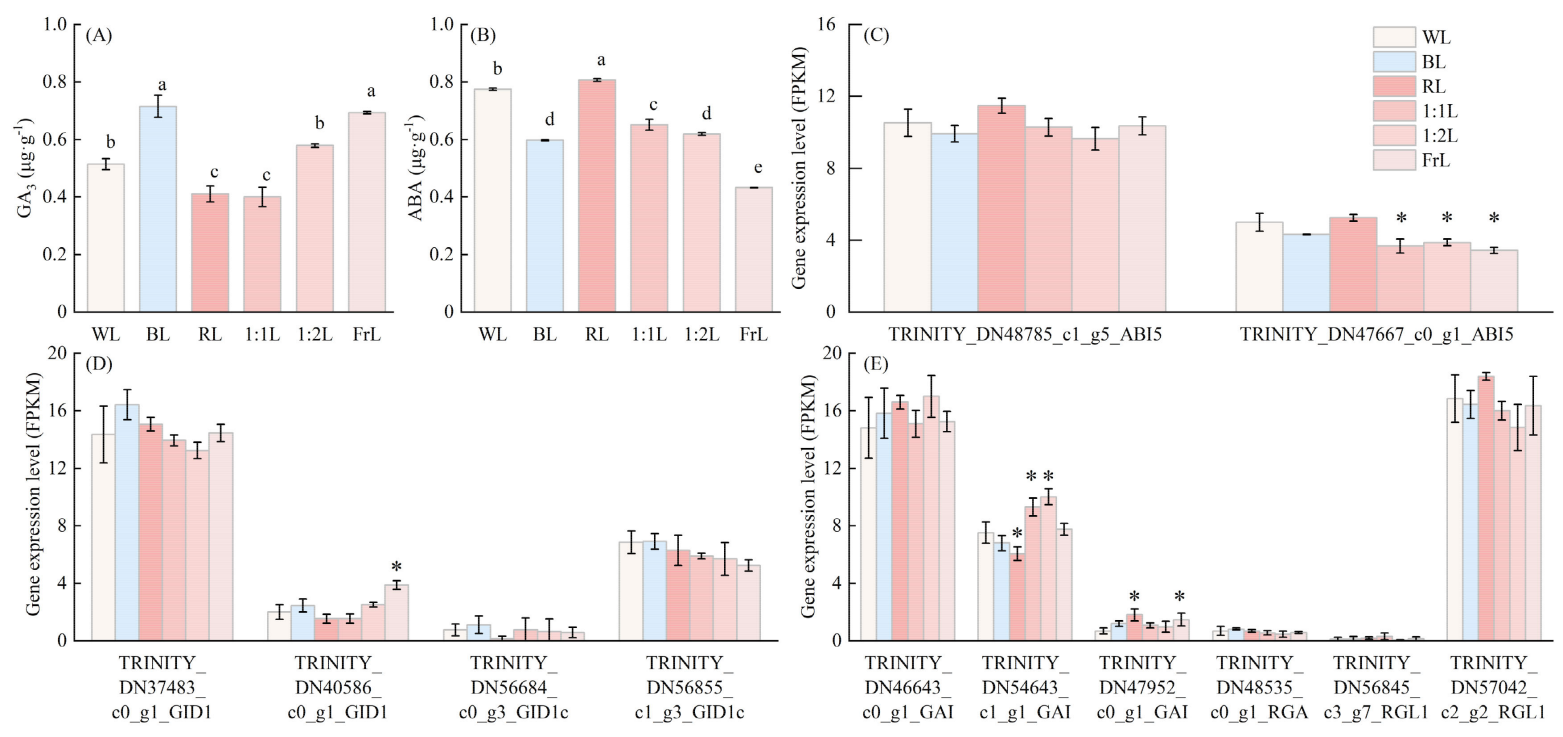
Effects of different light quality treatments on hormone levels and phytohormone signaling–related gene expression in *C. lanceolata* seedlings. **(A)** Gibberellin (GA3) content, **(B)** abscisic acid (ABA) content, **(C)** expression of ABI5, **(D)** expression of GID1, and **(E)** expression of DELLA protein genes. Data are presented as mean ± standard deviation. * indicates a significant difference compared with WL.

Hormone profiling revealed that GA3 content was highest under FrL and BL treatments but lowest under RL and 1:1L (**Figure 5A**). In contrast, ABA accumulation peaked under RL, whereas BL and FrL significantly reduced ABA levels (**Figure 5B**). Notably, GA3 content increased progressively with higher proportions of FrL, inversely correlated with ABA accumulation, indicating light quality–mediated GA–ABA antagonism.

For gene expression, two ABI5 transcripts (TRINITY_DN48785_c1_g5_ABI5 and TRINITY_DN47667_c0_g1_ABI5) were strongly upregulated by RL, consistent with elevated ABA content, while their expression was markedly suppressed under BL and FrL (**Figure 5C**). Among the GID1 homologs, most showed no significant differences, but TRINITY_DN40586 was significantly upregulated under FrL treatment (**Figure 5D**). Regarding DELLA proteins, GAI homologs (TRINITY_DN46643_c0_g1_GAI and TRINITY_DN47952_c0_g1_GAI) and the RGL1 homolog (TRINITY_DN57042_c2_ g2_RGL1) exhibited peak expression under RL, reinforcing the role of RL in activating GA signaling repressors (**Figure 5E**).

## 4 Discussion

This study elucidated how light quality regulates seedling development in *C. lanceolata* by integrating morphological, hormonal, and transcriptomic responses. In higher plants, light induced developmental plasticity is largely mediated by transcriptional reprogramming of light responsive genes (Ma et al., 2001; Moosavi-Nezhad et al., 2022). These molecular shifts are consistent with the observed changes in growth and hormone balance and provide mechanistic insight into how gymnosperm seedlings adjust to heterogeneous light environments (Martel and Qaderi, 2017; Dong et al., 2023; Gallo et al., 2024).

### 4.1 Light quality effects on morphological traits

Light quality produced clear and contrasting morphological outcomes. BL and FrL significantly promoted stem elongation, whereas RL inhibited elongation but enhanced root-collar diameter expansion. These contrasting responses indicate that spectral composition strongly influences seedling architecture, which may shape ecological strategies such as shade avoidance or stress tolerance. Similar responses have been reported in gymnosperms, although with species-specific differences. For instance, in Norway spruce, RL promotes elongation while BL suppresses it (OuYang et al., 2015). Such morphological divergence underscores the diversity of light responses in conifers and highlights the adaptive significance of spectral regulation.

### 4.2 Hormonal integration and antagonism between GAs and ABA

Light quality strongly modulated phytohormonal balance and thereby regulated the growth plasticity of *C. lanceolata* seedlings. Gibberellins (GAs) are central promoters of stem elongation, promoting skotomorphogenesis and suppressing photomorphogenesis, thereby leading to rapid stem elongation (Kayal et al., 2011; Lauria et al., 2024). In contrast, ABA antagonizes GAs activity and restricts elongation growth (Sun, 2010). Consistent with this paradigm, our results showed that GA_3_ levels were significantly higher under BL and FrL but lowest under RL, corresponding to enhanced elongation in BL and FrL and reduced elongation in RL. Conversely, ABA content and ABI5 expression peaked under RL, reinforcing the inhibitory effect of ABA in stem elongation (Lorrai et al., 2018). Previous studies likewise reported that RL decreases GA levels, whereas FrL substantially elevates them (Li et al., 2023; Wei et al., 2023, 2024).

At the molecular level, FrL significantly upregulated the GID1 homolog TRINITY_DN40586 (**Figure 5D**). RL strongly induced DELLA transcripts (e.g., GAI, RGL1), reinforcing its role in activating GA signaling repressors. This supports the involvement of a conserved GA–GID1–DELLA signaling module in *C. lanceolata*. The GA–GID1 receptor complex is known to promote rapid degradation of DELLA proteins (Sun, 2010), which act as negative regulators of GA signaling and thereby fine-tune plant sensitivity to GA (Castillon et al., 2007). In addition, PIF transcription factors further integrate light and hormone signaling by activating DELLA-related genes, thereby modulating seedling photomorphogenesis (de Lucas and Prat, 2014). Comparable patterns have been reported in poplar, where DELLA inhibitors accumulate under short-day conditions, reducing GA sensitivity, and in *Norway* sp*ruce*, where PIF3-LIKE1 and DELLA inhibitors are induced under BL (OuYang et al., 2015). These observations suggest that gymnosperms and angiosperms share conserved hormonal modules for integrating light and growth signals (Ruttink et al., 2007).

Moreover, ABA and GAs exhibited a strong negative correlation in this study, highlighting their antagonistic role in growth regulation (Sun, 2010; Liu and Hou, 2018). Compared with WL, ABA levels were markedly reduced under BL and FrL but elevated under RL, underscoring the role of spectral quality in hormone balance. Previous studies demonstrated that low red: far-red ratios suppress lateral bud growth but do not affect apical dominance, a response associated with differential ABA-related gene expression (Reddy et al., 2013). These dynamic hormonal shifts not only explain the contrasting growth responses of elongation versus dwarfing, but also provide molecular evidence for how light quality regulates growth plasticity in conifer seedlings (OuYang et al., 2015; Gallo et al., 2024).

### 4.3 Photoreceptor-mediated regulation and signal integration

The observed hormonal shifts were closely linked to photoreceptor-mediated signaling. RL markedly upregulated PHYB, consistent with activation of the phyB-PIF module, where active PHYB promotes PIF3 degradation through ubiquitination and suppresses elongation (Paik et al., 2019; Tripathi et al., 2019). PHYA expression remained consistently low across all light treatments (FPKM < 10), suggesting its instability under strong illumination. Under FrL, PIF3 expression increased, correlating with enhanced seedling height (**Figure 1A**) and indicating reliance on PIF3-mediated skotomorphogenic signaling. These patterns differ from Norway spruce, where RL promotes elongation and BL suppresses it (OuYang et al., 2015). BL and FrL also upregulated CRY1/CRY2 and COP1, indicating cryptochrome-mediated signaling contributes strongly to photomorphogenic regulation in conifers. Since cryptochromes act as both blue- and red-light receptors (Kasahara et al., 2004; Sellaro et al., 2009, 2025), this highlights potential crosstalk between BL and RL signaling in shaping seedling developmental.

### 4.4 Light quality and secondary metabolism

Beyond morphology and hormonal regulation, spectral variation also reprogrammed secondary metabolism in *C. lanceolata* seedlings. Light quality, as a critical environmental factor, not only regulates plant growth and development but also significantly influences the synthesis of secondary metabolites, thereby contributing to defense responses and light adaptation (Lauria et al., 2024). In this study, spectral shifts significantly regulated pathways associated with flavonoids, carotenoids, stilbenes, diarylheptanoids, and gingerol.

Under BL, the flavonoid biosynthesis pathway was significantly enriched, consistent with the role of flavonoids in antioxidant defense and photoprotection (Borbély et al., 2022). RL induced phenylpropanoid metabolism as well as pathways related to cutin, suberin, and wax deposition, suggesting structural reinforcement and enhanced stress tolerance. In contrast, FrL specifically enriched carotenoid and stilbenoid biosynthesis pathways, which may facilitate shade adaptation by improving light capture efficiency or modulating PHYA-dependent signaling (Sheerin and Hiltbrunner, 2017; Tan et al., 2022).

These findings are consistent with previous reports in gymnosperms. For example, in Norway spruce, BL enhances defense-related metabolism, particularly flavonoid accumulation, thereby improving stress resistance under variable light conditions (OuYang et al., 2015). The similarities between *C. lanceolata* and *Norway* sp*ruce* suggest that gymnosperms may share conserved strategies in leveraging secondary metabolism for photoprotection and environmental adaptation. Collectively, our results indicate that light spectra not only influence seedling growth and hormonal dynamics but also reshape metabolic pathways, equipping conifer seedlings with diverse adaptive mechanisms to thrive under heterogeneous light conditions.

While the RNA-seq data presented here provide a comprehensive overview of transcriptional responses to different light qualities, validation of key candidate genes using qRT-PCR would further strengthen these findings. Such validation experiments are planned as part of our ongoing research to confirm the observed expression patterns and elucidate the regulatory mechanisms underlying light-quality responses in *C. lanceolata.*

## 5 Conclusions

This study demonstrates that light quality profoundly regulates growth, transcriptional networks, and hormonal balance in *C. lanceolata* seedlings. Light treatments produced distinct morphological outcomes, with RL restraining elongation but promoting stem thickening, while BL and FrL enhanced stem elongation. Transcriptome analysis further revealed extensive differential gene expression, in which PIFs, HY5, and COP1 acted as central regulators mediating light responses. Hormone profiling showed clear antagonistic dynamics between GA and ABA under different spectra. RL elevated ABA accumulation, whereas BL and FrL increased GA_3_ levels and reduced ABA, thereby shaping growth plasticity. In addition, secondary metabolic pathways were differentially activated, with BL stimulating flavonoid biosynthesis, RL enhancing phenylpropanoid and cell wall pathways, and FrL inducing carotenoid and stilbenoid metabolism. These findings provide mechanistic insights into how light signaling, hormonal crosstalk, and metabolic reprogramming shape gymnosperm morphogenesis. By clarifying the molecular basis of light quality–dependent growth responses in *C. lanceolata*, this work provides a foundation for optimizing nursery light regimes and improving regeneration practices in subtropical conifer plantations.

## Data Availability

The data of this study are deposited in the National Genomics Data Center (NGDC), repository link: https://ngdc.cncb.ac.cn, accession number PRJCA060854.

## References

[B1] Borbély P. Gasperl A. Pálmai T. Ahres M. Asghar M. A. Galiba G. . (2022). Light intensity- and spectrum-dependent redox regulation of plant metabolism. Antioxidants 11, 1311. doi: 10.3390/antiox11071311, PMID: 35883801 PMC9312225

[B2] Boros I. F. Székely G. Balázs L. Csambalik L. Sipos L. (2023). Effects of LED Lighting Environments on Lettuce (*Lactuca sativa* L.) in PFAL systems – A review. Scientia Hortic. 321, 112351. doi: 10.1016/j.scienta.2023.112351, PMID: 41909469

[B3] Castillon A. Shen H. Huq E. (2007). Phytochrome interacting factors: central players in phytochrome-mediated light signaling networks. Trends Plant Sci. 12, 514–521. doi: 10.1016/j.tplants.2007.10.001, PMID: 17933576

[B4] Chen M. Xu S. Ye Y. Lin K. Lan W. Cao G. (2025). Integrative analysis of transcriptome and metabolome reveals light quality-mediated regulation of adventitious shoot proliferation in chinese fir. Forests 16, 486. doi: 10.3390/f16030486, PMID: 41725453

[B5] de Lucas M. Prat S. (2014). PIFs get BR right: PHYTOCHROME INTERACTING FACTORs as Integrators of Light and Hormonal Signals. New Phytol. 202, 1126–1141. doi: 10.1111/nph.12725, PMID: 24571056

[B6] Dickson A. Leaf A. L. Hosner J. F. (1960). Quality appraisal of white spruce and white pine seedling stock in nurseries. Forestry Chronicle 36, 10–13. doi: 10.5558/tfc36010-1

[B7] Doneva D. Pál M. Szalai G. Vasileva I. Brankova L. Misheva S. . (2024). Manipulating the light spectrum to increase the biomass production, physiological plasticity and nutritional quality of eruca sativa L. Plant Physiol. Biochem. 217, 109218. doi: 10.1016/j.plaphy.2024.109218, PMID: 39461053

[B8] Dong T. Zhang P. Hakeem A. Liu Z. Su L. Ren Y. . (2023). Integrated transcriptome and metabolome analysis reveals the physiological and molecular mechanisms of grape seedlings in response to red, green, blue, and white led light qualities. Environ. Exp. Bot. 213, 105441. doi: 10.1016/j.envexpbot.2023.105441, PMID: 41909469

[B9] Gallo A. E. Christophe A. Poupard M. Boulord R. Rolland G. Prieto J. A. . (2024). Effects of acclimation to long-term shading on photosynthesis in grapevines: roles of non-structural carbohydrates and stomatal conductance. Physiologia Plantarum 176, e14636. doi: 10.1111/ppl.14636, PMID: 39604291

[B10] García-Martinez J. L. Gil J. (2001). Light regulation of gibberellin biosynthesis and mode of action. J. Plant Growth Regul. 20, 354–368. doi: 10.1007/s003440010033, PMID: 11986761

[B11] Gómez-Ocampo G. Cascales J. Medina-Fraga A. L. Ploschuk E. L. Mantese A. I. Crocco C. D. . (2023). Transcriptomic and physiological shade avoidance responses in potato (*Solanum tuberosum*) plants. Physiologia Plantarum 175, e13991. doi: 10.1111/ppl.13991, PMID: 37616016

[B12] Huber M. Nieuwendijk N. M. Pantazopoulou C. K. Pierik R. (2021). Light signalling shapes plant–plant interactions in dense canopies. Plant, Cell & Environment 44 (4), 1014–1029. doi: 10.1111/pce.13912, PMID: 33047350 PMC8049026

[B13] Kang H. X. Han J. Y. Mu X. H. Chen J. Zhang L. M. Tang Y. H. (2024). Efficient absorption of green light by the canopy of a monoculture coniferous forest. Agric. For. Meteorology 351, 110006. doi: 10.1016/j.agrformet.2024.110006, PMID: 41909469

[B14] Kasahara M. Kagawa T. Sato Y. Kiyosue T. Wada M. (2004). Phototropins mediate blue and red light-induced chloroplast movements in *physcomitrella patens*. Plant Physiol. 135, 1388–1397. doi: 10.1104/pp.104.042705, PMID: 15247376 PMC519056

[B15] Kayal W. E. Allen C. C. G. Ju CJ-T. Adams E. King-Jones S. Zaharia L. I. . (2011). Molecular events of apical bud formation in white spruce, *picea glauca*. Plant Cell Environ. 34, 480–500. doi: 10.1111/j.1365-3040.2010.02257.x, PMID: 21118421

[B16] Lauria G. Ceccanti C. Lo Piccolo E. El Horri H. Guidi L. Lawson T. . (2024). Metabolight”: how light spectra shape plant growth, development and metabolism. Physiologia Plantarum 176, e14587. doi: 10.1111/ppl.14587, PMID: 39482564

[B17] Li W. X. Fang Q. T. Han Q. O. Huang H. H. Zheng X. Q. Lu J. L. . (2025). Different performance of tea plants to shade based on key metabolites and transcriptome profiles: case study of cultivars longjing 43 and yabukita. Physiologia Plantarum 177, e70103. doi: 10.1111/ppl.70103, PMID: 39905973

[B18] Li Y. Jiang H. Gao M. He R. Liu X. Su W. . (2023). Far-red-light-induced morphology changes, phytohormone, and transcriptome reprogramming of chinese kale (*Brassica alboglabra* bailey). Int. J. Mol. Sci. 24, 5563. doi: 10.3390/ijms24065563, PMID: 36982639 PMC10053878

[B19] Liu X. Hou X. (2018). Antagonistic regulation of ABA and GA in metabolism and signaling pathways. Frontier Plant Sci. 9, 251. doi: 10.3389/fpls.2018.00251, PMID: 29535756 PMC5834473

[B20] Liu Q. Huang Z. Ma X. Tigabu M. Xing X. Jin S. . (2022). Phenotypic plasticity of *cunninghamia lanceolata* (Lamb.) hook. Seedlings in response to varied light quality treatments. Forests 13, 201. doi: 10.3390/f13020201, PMID: 41725453

[B21] Liu B. Liu Q. Daryanto S. Guo S. Huang Z. Wang Z. . (2018). Responses of Chinese fir and *Schima superba* Seedlings to Light Gradients: Implications for the Restoration of Mixed Broadleaf-Conifer Forests from Chinese Fir Monocultures. For. Ecol. Manage. 419–420, 51–57. doi: 10.1016/j.foreco.2018.03.033, PMID: 41909469

[B22] Liu H. Son J. E. Niu G. Li Q. (2024). Editorial: growth and quality formation regulated by light in horticulture plants. Frontier Plant Sci. 15, 1414970. doi: 10.3389/fpls.2024.1414970, PMID: 38817932 PMC11137296

[B23] Lorrai R. Boccaccini A. Ruta V. Possenti M. Costantino P. Paola V. (2018). Abscisic acid inhibits hypocotyl elongation acting on gibberellins, DELLA proteins and auxin. AoB Plants 10, ply061. doi: 10.1093/aobpla/ply061, PMID: 30386544 PMC6204436

[B24] Love M. I. Huber W. Anders S. (2014). Moderated estimation of fold change and dispersion for RNA-seq data with DESeq2. Genome Biology 15 (12), 550. doi: 10.1186/s13059-014-0550-8, PMID: 25516281 PMC4302049

[B25] Ma L. Li J. Qu L. Hager J. Chen Z. Zhao H. . (2001). Light control of arabidopsis development entails coordinated regulation of genome expression and cellular pathways. Plant Cell 13, 2589–2607. doi: 10.1105/tpc.010229, PMID: 11752374 PMC139475

[B26] Martel A. B. Qaderi M. M. (2017). Light quality and quantity regulate aerobic methane emissions from plants. Physiologia Plantarum 159, 313–328. doi: 10.1111/ppl.12514, PMID: 27717171

[B27] Matsuo S. Nanya K. Imanishi S. Honda I. Goto E. (2019). Effects of blue and red lights on gibberellin metabolism in tomato seedlings. Hortic. J. 88, 76–82. doi: 10.2503/hortj.UTD-005

[B28] Montgomery R. Chazdon R. J. (2002). Light gradient partitioning by tropical tree seedlings in the absence of canopy gaps. Oecologia 131, 165–174. doi: 10.1007/s00442-002-0872-1, PMID: 28547683

[B29] Moosavi-Nezhad M. Alibeigi B. Estaji A. Gruda N. S. Aliniaeifard S. (2022). Growth, Biomass Partitioning, and Photosynthetic Performance of Chrysanthemum Cuttings in Response to Different Light Spectra. Plants 11 (23), 3337. doi: 10.3390/plants11233337, PMID: 36501376 PMC9735900

[B30] Navidad H. Fløistad I. S. Olsen J. E. Torre S. (2020). Subalpine Fir (*Abies laciocarpa*) and Norway Spruce (*Picea abies*) Seedlings Show Different Growth Responses to Blue Light. Agronomy 10, 712. doi: 10.3390/agronomy10050712, PMID: 41725453

[B31] OuYang F. Mao J.-F. Wang J. Zhang S. Li Y. (2015). Transcriptome analysis reveals that red and blue light regulate growth and phytohormone metabolism in Norway spruce [*Picea abies* (L.) karst. PloS One 10, e0127896. doi: 10.1371/journal.pone.0127896, PMID: 26237749 PMC4523189

[B32] Paik I. Chen F. Ngoc Pham V. Zhu L. Kim J.-I. Huq E. (2019). A phyB-PIF1-SPA1 kinase regulatory complex promotes photomorphogenesis in arabidopsis. Nat. Commun. 10, 4216. doi: 10.1038/s41467-019-12110-y, PMID: 31527679 PMC6746701

[B33] Reddy S. K. Holalu S. V. Casal J. J. Finlayson S. A. (2013). Abscisic acid regulates axillary bud outgrowth responses to the ratio of red to far-red light. Plant Physiol. 163, 1047–1058. doi: 10.1104/pp.113.221895, PMID: 23929720 PMC3793024

[B34] Ruberti I. Sessa G. Ciolfi A. Possenti M. Carabelli M. Morelli G. (2012). Plant adaptation to dynamically changing environments: the shade avoidance response. Biotechnol. Adv. 30, 1047–1058. doi: 10.1016/j.biotechadv.2011.08.014, PMID: 21888962

[B35] Ruttink T. Arend M. Morreel K. Storme V. Rombauts S. Fromm J. . (2007). A molecular timetable for apical bud formation and dormancy induction in poplar. Plant Cell 19, 2370–2390. doi: 10.1105/tpc.107.052811, PMID: 17693531 PMC2002631

[B36] Sellaro R. Durand M. Aphalo P. J. Casal J. J. (2025). Making the most of canopy light: shade avoidance under a fluctuating spectrum and irradiance. J. Exp. Bot. 76, 712–729. doi: 10.1093/jxb/erae334, PMID: 39101508 PMC11805590

[B37] Sellaro R. Hoecker U. Yanovsky M. Chory J. Casal J. J. (2009). Synergism of red and blue light in the control of arabidopsis gene expression and development. Curr. Biol. 19, 1216–1220. doi: 10.1016/j.cub.2009.05.062, PMID: 19559617 PMC2730174

[B38] Sheerin D. J. Hiltbrunner A. (2017). Molecular mechanisms and ecological function of far-red light signalling. Plant Cell Environ. 40, 2509–2529. doi: 10.1111/pce.12915, PMID: 28102581

[B39] Sun T. (2010). Gibberellin-GID1-DELLA: A pivotal regulatory module for plant growth and development. Plant Physiol. 154, 567–570. doi: 10.1104/pp.110.161554, PMID: 20921186 PMC2949019

[B40] Tan T. Li S. Fan Y. Wang Z. Ali Raza M. Shafiq I. . (2022). Far-red light: A regulator of plant morphology and photosynthetic capacity. Crop J. 10, 300–309. doi: 10.1016/j.cj.2021.06.007, PMID: 41909469

[B41] Toman E. Käs M. D. Taubert B. Moritz M. Qi J. Bolle C. (2024). Photosynthetic and plastid performance effects of photoreceptors. J. Mitochondria Plastids Endosymbiosis 2, 2330972. doi: 10.1080/28347056.2024.2330972, PMID: 41909888

[B42] Tripathi S. Hoang Q. T. N. Han Y.-J. Kim J.-I. (2019). Regulation of photomorphogenic development by plant phytochromes. Int. J. Mol. Sci. 20, 6165. doi: 10.3390/ijms20246165, PMID: 31817722 PMC6941077

[B43] Van Brenk J. B. Hendriks L. Rei A. Marcelis L. F. M. Verdonk J. C. (2025). Dynamic application of high and low red: blue ratios during lettuce development shifts growth and metabolite allocation. Physiologia Plantarum 177, e70456. doi: 10.1111/ppl.70456, PMID: 40808356 PMC12351208

[B44] Wan T. Gong Y. Liu Z. Zhou Y. Dai C. Wang Q. (2022). Evolution of complex genome architecture in gymnosperms. GigaScience 11, giac078. doi: 10.1093/gigascience/giac078, PMID: 35946987 PMC9364684

[B45] Wang X. Chen G. Meng F. Zhu Q. Fan F. Cao S. (2022). Effects of light quality on photosynthetic physiology and pigment accumulation of chinese fir seedlings. J. Cent. South Univ. Forestry Technol. 12 82–90, 111.

[B46] Wang Z. Luo H. Liu B. Song S. Zhang X. Song Y. . (2024). Response of morphological plasticity of *quercus variabilis* seedlings to different light quality. Forests 15, 2153. doi: 10.3390/f15122153, PMID: 41725453

[B47] Wei H. Hauer R. J. Chen G. Chen X. He X. (2019). Growth, nutrient assimilation, and carbohydrate metabolism in korean pine (*Pinus koraiensis*) seedlings in response to light spectra. Forests 11, 44. doi: 10.3390/f11010044, PMID: 41725453

[B48] Wei C. Luo G. Jin Z. Li J. Li Y. (2024). Physiological and structural changes in leaves of *platycrater arguta* seedlings exposed to increasing light intensities. Plants 13, 1263. doi: 10.3390/plants13091263, PMID: 38732478 PMC11085374

[B49] Wei Y. Wang S. Yu D. (2023). The role of light quality in regulating early seedling development. Plants 12, 2746. doi: 10.3390/plants12142746, PMID: 37514360 PMC10383958

[B50] Weyers J. D. B. Paterson N. W. (2001). Plant hormones and the control of physiological processes. New Phytol. 152, 375–407. doi: 10.1046/j.0028-646X.2001.00281.x, PMID: 33862994

[B51] Wirth R. Weber B. Ryel R. J. (2001). Spatial and temporal variability of canopy structure in a tropical moist forest. Acta Oecologica 22, 235–244. doi: 10.1016/S1146-609X(01)01123-7, PMID: 41908905

[B52] Yang Y. Wang L. Yang Z. Xu C. Xie J. Chen G. . (2018). Large ecosystem service benefits of assisted natural regeneration. J. Geophysical Research: Biogeosciences 123, 676–687. doi: 10.1002/2017JG004267, PMID: 41889077

