# Supplementary material for: Growth, phytohormone and transcriptome responses of Cunninghamia lanceolata seedlings to different light qualities
Source: Front Plant Sci. 2026 Apr 7;17:1765282. doi: 10.3389/fpls.2026.1765282 (PMC13095536; doi:10.3389/fpls.2026.1765282)
Supplement: Supplementary file 1 [file DataSheet1.docx]

Supplementary Material

**
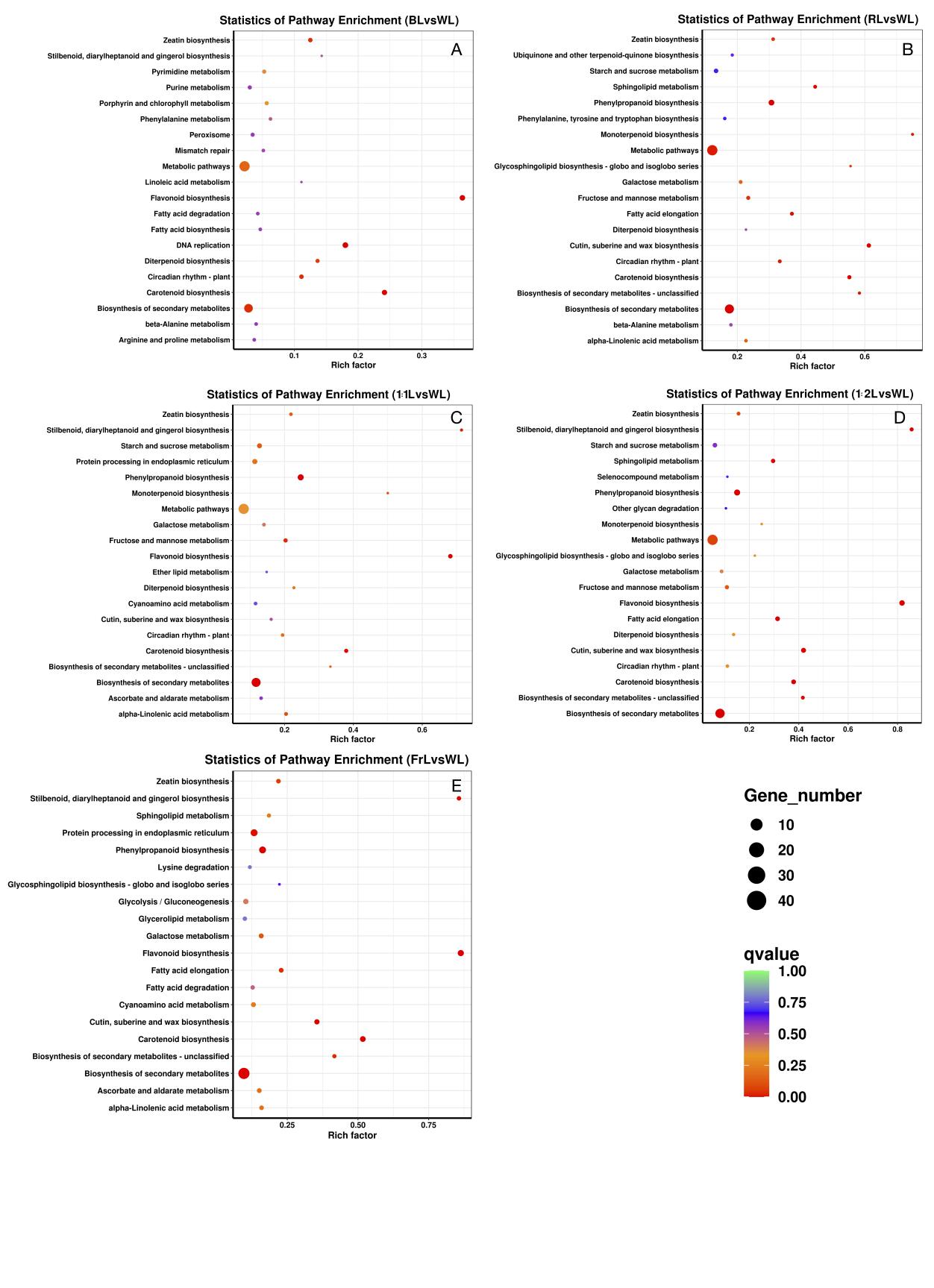
**

**Supplementary Figure 1.** KEGG enrichment analysis of differentially expressed genes (DEGs) in C. lanceolata seedlings under different light quality treatments. The y-axis shows significantly enriched pathways, and the x-axis represents the rich factor, defined as the ratio of DEGs to the total number of annotated genes in a pathway. Dot size reflects the number of DEGs, and color gradients indicate the adjusted q-values, with red representing higher significance.
