# Supplementary material for: Growth, phytohormone and transcriptome responses of Cunninghamia lanceolata seedlings to different light qualities
Source: Front Plant Sci. 2026 Apr 7;17:1765282. doi: 10.3389/fpls.2026.1765282 (PMC13095536; doi:10.3389/fpls.2026.1765282)
Supplement: Supplementary file 2 [file Table1.docx]

Supplementary Material

**Table S1**. Summary of sequencing data quality after filtering in *C. lanceolata* seedlings grown under different light quality treatments.

| Sample | Total Raw Reads | Total Clean Reads | Total Clean Bases (G) | Error Rate (%) | Q20  (%) | Q30  (%) | GC Content (%) |
| --- | --- | --- | --- | --- | --- | --- | --- |
| WL | 64192250 | 62439999 | 9.43 | 0.02 | 98.73 | 96.09 | 44.38 |
| RL | 56993230 | 55343472 | 8.36 | 0.02 | 98.77 | 96.20 | 44.27 |
| BL | 56937113 | 55000070 | 8.30 | 0.02 | 98.73 | 96.06 | 44.40 |
| 1:1L | 65047081 | 62589147 | 9.45 | 0.02 | 98.683 | 96.00 | 44.40 |
| 1:2L | 66269445 | 63909673 | 9.65 | 0.02 | 98.74 | 96.07 | 44.57 |
| FrL | 78694550 | 76264521 | 11.5 | 0.02 | 98.73 | 96.03 | 44.50 |
